# Impact of multiparametric MRI and prostate biopsies on anxiety and quality of life in men with suspected prostate cancer

**DOI:** 10.1002/bco2.70087

**Published:** 2025-10-17

**Authors:** Esther H. J. Hamoen, Bas Israël, Marloes van der Leest, Erik B. Cornel, O. Sjoerd Klaver, Rianne J. Hendriks, Jeroen Veltman, Inge M. van Oort, Gerjon Hannink, J. Alfred Witjes, Jelle O. Barentsz, Maroeska M. Rovers

**Affiliations:** ^1^ Department of Medical Imaging Radboud University Medical Center Nijmegen The Netherlands; ^2^ Department of Urology Radboud University Medical Center Nijmegen The Netherlands; ^3^ Department of Urology Ziekenhuis Groep Twente Hengelo The Netherlands; ^4^ Department of Urology Erasmus Medical Center Rotterdam The Netherlands; ^5^ Department of Radiology Ziekenhuis Groep Twente Hengelo The Netherlands

**Keywords:** anxiety, biopsy, diagnosis, magnetic resonance imaging, prostate cancer, quality of life

## Abstract

**Objectives:**

To study the impact of the new MRI pathway and conventional transrectal ultrasound‐guided systematic biopsies (TRUSGB) on anxiety and HRQoL in men with suspected PCa.

**Materials and methods:**

A secondary analysis was performed of a randomized clinical trial including 626 biopsy‐naïve patients. All patients underwent mpMRI and TRUSGB. Men with suspicious lesions on mpMRI underwent MRGB prior to TRUSGB. Anxiety was measured by State–Trait Anxiety Inventory‐Trait Scale (STAI‐6), completed at baseline, directly after mpMRI, MRGB, TRUSGB, after two/three weeks, and six months. HRQoL was measured by EuroQol (EQ‐5D‐5L), European Organization for Research and Treatment of Cancer Quality of Life Questionnaire Core 30 (QLQ‐C30) and Prostate Cancer Module (QLQ‐PR25). Outcomes were compared between patients that underwent mpMRI and TRUSGB and patients that underwent mpMRI, MRGB and TRUSGB. Differences were considered relevant if the 95% confidence interval exceeded the minimal important clinical difference.

**Results:**

No relevant differences were seen in anxiety scores and generic HRQoL at different time points in patients that underwent mpMRI, TRUSGB and MRGB compared to patients that underwent mpMRI and TRUSGB. Patients that underwent mpMRI, MRGB and TRUSGB reported lower incontinence aid and hormonal treatment‐related symptom scores after 6 months compared to patients that underwent mpMRI and TRUSGB.

**Conclusions:**

In men suspected of PCa, no differences were observed in anxiety levels or generic HRQoL scores across different diagnostic pathways. However, lower PCa‐specific HRQoL subscores were noted in patients who underwent mpMRI, MRGB and TRUSGB.

## 1 INTRODUCTION

Prostate cancer (PCa) is one of the most common cancers among men and is the third leading cause of cancer death in men in Europe.^1^ Traditionally, PCa was diagnosed by systematic transrectal ultrasound‐guided biopsies (TRUSGB). Compared with TRUSGB, mpMRI has been reported to reduce the detection of clinically insignificant PCa (cisPCa) while increasing the detection of clinically significant PCa (csPCa). Since 2020, the diagnostic pathway in the PCa guidelines has changed. Patients with suspected csPCa are now recommended to undergo an MRI followed by targeted biopsy if the MRI is suspicious. The opportunity to selectively localize csPCa enables MR‐directed biopsy and, in so doing, fewer cores. If mpMRI is non‐suspicious, immediate TRUSGB can be safely avoided.^2^

Although the impact of cancer diagnosis and treatment on health‐related quality of life (HRQoL) has gained more attention, few studies have examined the diagnostic experience of patients with an unknown diagnosis or with a non‐cancer result.^3^ Studies in patients suspected of different types of cancer showed that many experience considerable distress before and during the diagnostic process irrespective of the final diagnosis. Anxiety levels, however, decrease within 30 days in case of a benign diagnosis.^3^, ^4^, ^5^

After changing standard procedures, it may be relevant to evaluate the impact of these procedures on HRQoL. The potential advantages of an MRI pathway for patients (i.e. to improve the detection of csPCa and use fewer cores) could lead to a positive effect on HRQoL and reduce anxiety compared to TRUSGB. So far, the impact of TRUSGB and targeted ultrasound‐guided biopsy on patients' anxiety has been studied by Chesnut et al.^6^ However, mpMRI and MR‐guided biopsy (MRGB) were not evaluated. Shanker et al. measured anxiety and general HRQoL in active surveillance patients comparing TRUSGB to MRI.^7^ Main results of this study were lower pre‐procedure anxiety scores in the MRI pathway versus the TRUSGB pathway and higher pain scores during the procedure in the TRUSGB cohort. One other study found comparable HRQoL results in patients who underwent MRGB and those with TRUSGB.^8^ Both studies, however, only used general HRQoL questionnaires rather than PCa‐specific HRQoL questionnaires.

**FIGURE 1:**
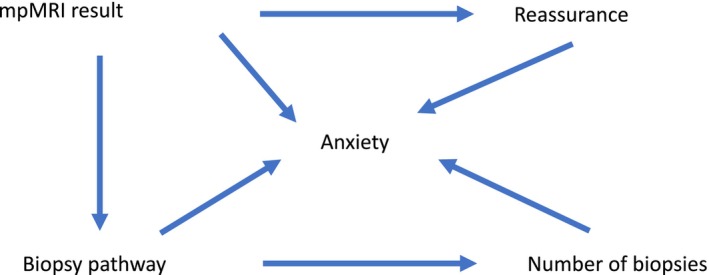
**Conceptual framework to illustrate the possible mechanisms by which biopsy route may influence anxiety.** *mpMRI = multiparametric magnetic resonance imaging,*

Figure 1 shows a diagram with a conceptual framework to illustrate the possible mechanisms by which the biopsy route may influence anxiety. Our hypothesis was that patients who underwent mpMRI and TRUSGB would show lower anxiety scores and better HRQoL scores compared to those who underwent mpMRI, MRGB and TRUSGB. This was based on the assumption that a “non‐suspicous” mpMRI and undergoing fewer prostate biopsies would provide greater reassurance.

We performed a secondary analysis of a prospective study in which we described and compared anxiety, generic and PCa‐specific HRQoL outcomes in patients who underwent mpMRI and TRUSGB and patients who underwent mpMRI, MRGB and TRUSGB.

## 2 MATERIALS AND METHODS

This prospective multicentre study was performed within the 4 M study and was conducted from February 2015 to February 2018. The full study protocol and results have been described previously.^9^ Eligible men were biopsy‐naïve, aged 50–75 yr with a PSA value of ≥3 ng/ml. A total of 699 men were included from four institutions in the Netherlands (Radboudumc, Maasstad Hospital, Andros Men's Health Clinic, Ziekenhuis Groep Twente). The study was registered in the Dutch Trial Register (NTR5555).

### 2.1 MRI examination and image analysis

All patients underwent mp‐MRI examinations on 3‐Tesla MRI scanners (Magnetom Skyra; Siemens Healthineers, Germany) with pelvic phased‐array coils, according to PI‐RADS v2 standards. The protocol consisted of T2‐weighted imaging, diffusion‐weighted imaging and dynamic contrast‐enhanced imaging.^9^

### 2.2 Systematic and targeted biopsy

Men with a suspicious lesion on mp‐MRI examination (i.e., PI‐RADS 3–5) underwent a two‐ to four‐core in‐bore MRGB of each suspicious lesion using a transrectal in‐bore MR biopsy device (Invivo, Gainsville, FL, USA). Then, blinded to MRI findings, a 12‐core TRUSGB was performed. Both procedures used 18G needles with a sampling length of 17 mm. TRUSGB was performed according to standard international guidelines. Where a lesion was visible at TRUS, it was targeted by using the core for the relevant prostate zone. Men with a nonsuspicious mp‐MRI underwent TRUSGB only.

### 2.3 Histopathology

All biopsies were reviewed at the central centre by an experienced uropathologist, independent of the results of the nonuniversity pathologists and the mpMRI results. TRUSGB and MRGB of each patient were evaluated separately and blinded to individual results. For cores containing cancer, grade group and Gleason score (GS) were determined using 2014 International Society of Urologic Pathology criteria.^10^ csPCa was defined as the highest GS ≥ 3 + 4, detected by MRGB or TRUSGB.

### 2.4 Outcome measures

Outcomes of this study were anxiety, generic HRQoL, cancer‐specific HRQoL and PCa‐specific HRQoL measured by digital‐based questionnaires.

#### 2.4.1 Anxiety

Anxiety was assessed with the six‐item State–Trait Anxiety Inventory (STAI‐6). Total scores range from 20 to 80, with 80 indicating maximum anxiety.^11^

#### 2.4.2 Generic HRQoL

Generic HRQoL was measured with EQ‐5D‐5L. The first part of the EQ‐5D is a questionnaire consisting of five questions on dimensions mobility, self‐care, usual activities, pain/discomfort and anxiety/depression on a five‐point Likert scale. This is transformed into a health utility (index value).^12^ The second part is a visual analogue scale (VAS), which records the patient's self‐rated health on a vertical scale.

#### 2.4.3 Cancer‐specific and prostate‐specific HRQoL

Cancer‐specific HRQoL was measured by the European Organization for Research and Treatment of Cancer (EORTC) Quality of Life Questionnaire Core 30 (QLQ‐C30).^13^ Five functional scales measure physical, role, emotional, cognitive and social functioning along with one global QoL scale. Three symptom scales describe fatigue, nausea/vomiting and pain. Six single items concern dyspnea, insomnia, appetite loss, constipation, diarrhoea and financial difficulties.

The EORTC Prostate Cancer Module (QLQ‐PR25) was used to measure PCa‐specific HRQoL.^14^ QLQ‐PR25 consists of five multi‐item scales: urinary symptoms, bowel symptoms, hormonal treatment‐related symptoms, sexual activity and sexual functioning (conditional). Bother due to the use of an incontinence aid was measured by a single item (conditional). All QLQ‐C30 and QLQ‐PR25 scales were linearly transformed to a scale from 0 to 100.

**FIGURE 2:**
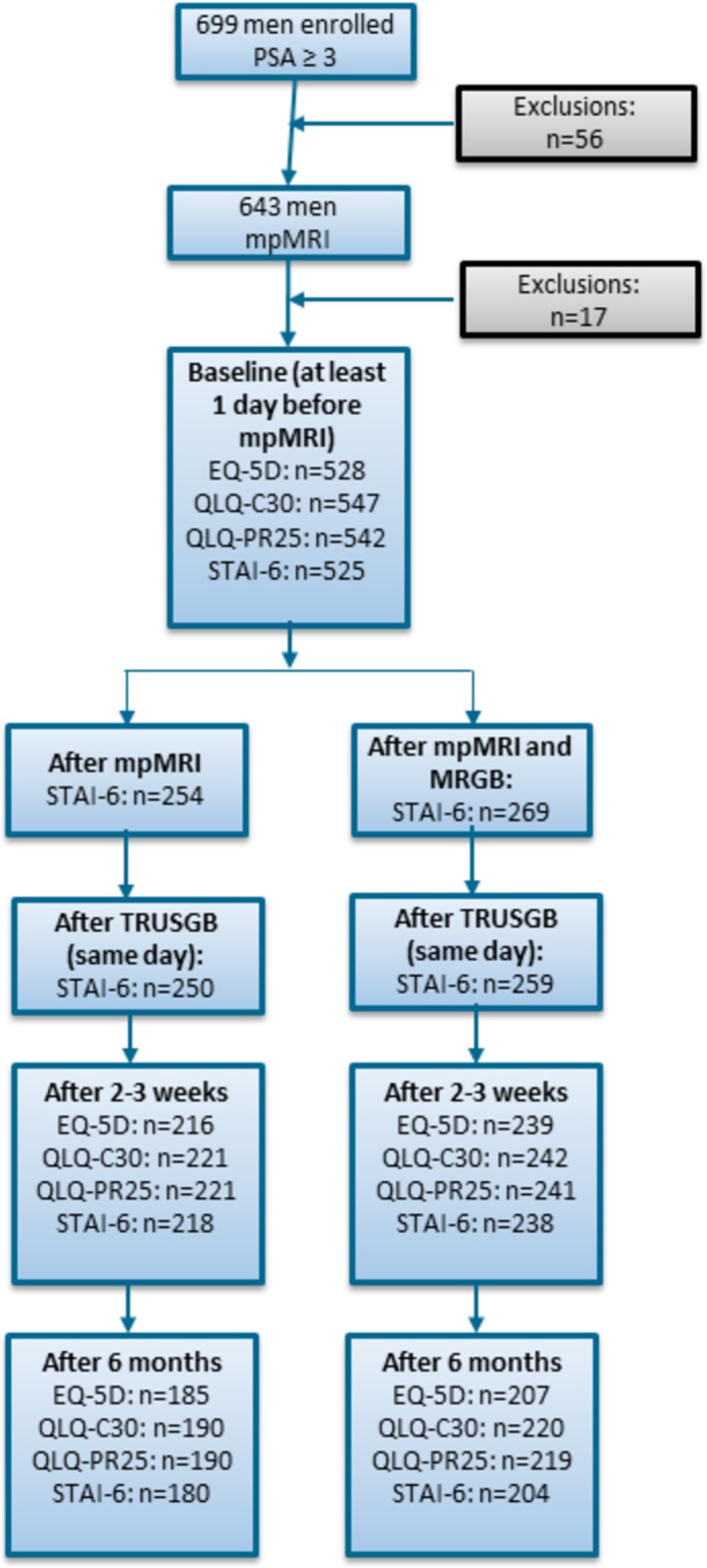
**Timeline of the study.** *PSA = prostate specific antigen, mpMRI = multiparametric magnetic resonance imaging, MRGB = in‐bore MR‐guided biopsies, TRUSGB = systematic transrectal ultrasound guided biopsies, equation 5D = EuroQol five dimensions, QLQ‐C30) = the European Organization for Research and Treatment of cancer quality of life questionnaire Core 30, QLQ‐PR25 = prostate cancer module of the European Organization for Research and Treatment of cancer quality of life questionnaire, STAI = state–trait anxiety inventory.*

A timeline of measurements and flow chart of the study are shown in Figure 2. At baseline, EQ‐5D, QLQ‐C30, QLQ‐PR25 and STAI‐6 were measured. Directly after mp‐MRI and/or MRGB, and on the same day after TRUSGB, STAI‐6 was measured again. After 2–3 weeks and after 6 months, EQ‐5D, QLQ‐C30, QLQ‐PR25 and STAI‐6 were repeatedly measured. Outcomes were described and compared for patients who underwent mpMRI and TRUSGB and patients who underwent mpMRI, MRGB and TRUSGB.

### 2.5 Statistical analysis

Scores were reported as means (standard deviations, SDs). Data displayed in graphs are mean scores (SD) of STAI‐6, QLQ‐C30 and QLQ‐PR25. Minimally important clinical differences (MICDs) were used to assess clinically relevant changes over time or differences between groups (mpMRI and TRUSGB versus mpMRI, MRGB and TRUSGB). Thresholds for MICDs are shown in Table S1. For EQ‐5D‐VAS and QLQ‐C30, we used available MICDs.^15^, ^16^ As no MICD was available for QLQ‐PR25 and STAI‐6, we used 0.5 SD of our baseline results as MICD for these scales.^17^ Scores changes were calculated as mean (95% confidence interval, CI); group differences were calculated as mean difference (95%CI). Differences were considered clinically relevant if the entire 95 % CI exceeded MICD.

STAI‐6 and HRQoL were compared in patients who underwent mpMRI followed by TRUSGB, and patients who underwent mpMRI followed by MRGB and TRUSGB. Furthermore, scores of patients in whom PCa was detected were compared to scores of patients without PCa. All analyses were performed with SPSS, version 25 (IBM, Armonk, NY, USA).

## 3 RESULTS

For final analysis, 626 men were included. All 626 patients underwent mpMRI and TRUSGB, and 317 underwent MRGB (Figure 2).

### 3.1 Anxiety

**FIGURE 3:**
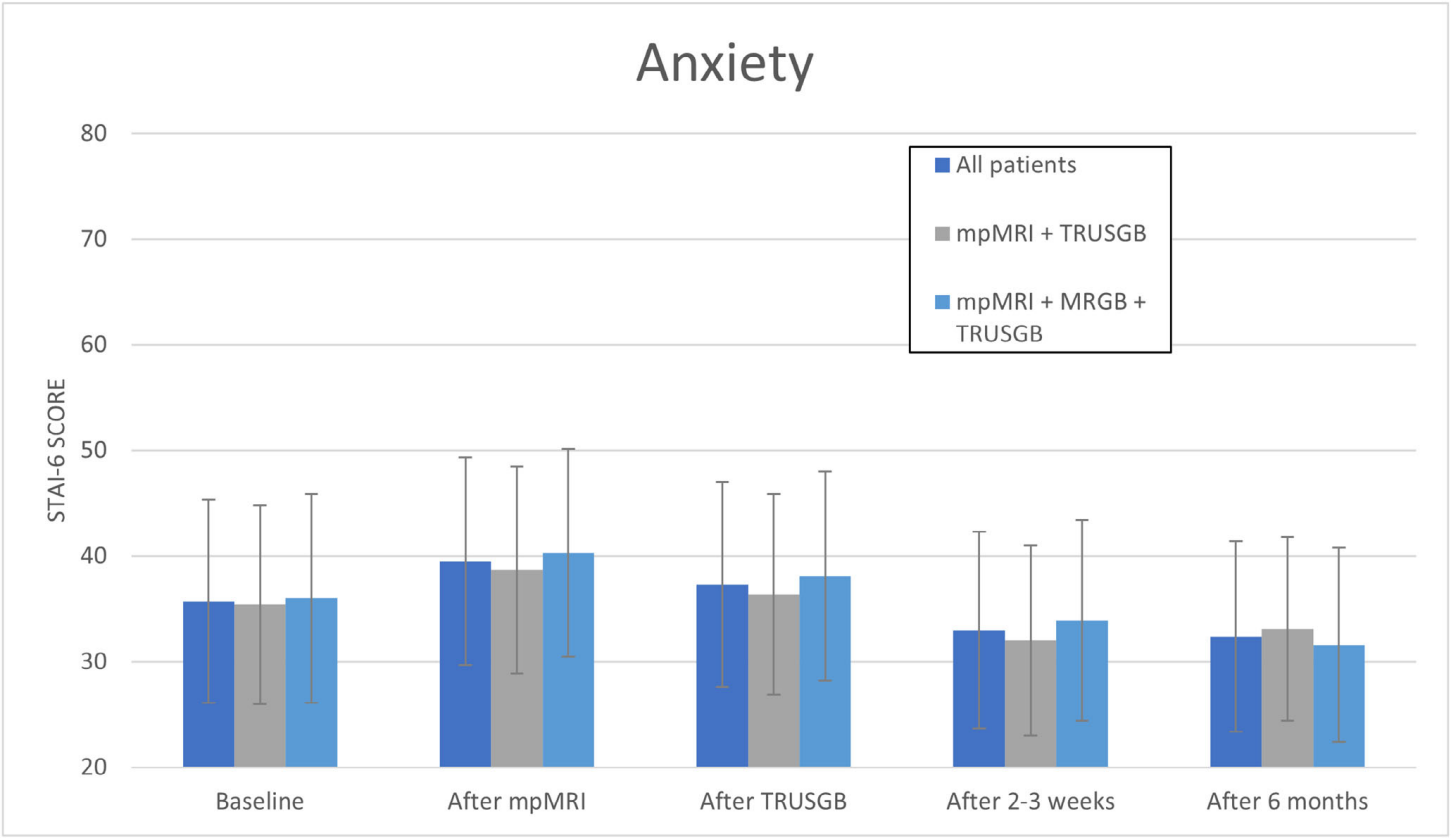
**State–trait anxiety inventory (STAI‐6) scores at different time points. Scores range from 20 to 80; a higher score represents a higher level of anxiety.** *mpMRI = multiparametric magnetic resonance imaging, MRGB = in‐bore MR‐guided biopsies, TRUSGB = systematic transrectal ultrasound‐guided biopsies, SD = standard deviation.*

Anxiety scores were highest after mpMRI (40.0 ± 9.8) and declined to 32 ± 9.0 after six months (Figure 3). No clinically relevant differences were found in patients who underwent mpMRI, MRGB and TRUSGB compared to patients who underwent mpMRI and TRUSGB (Table S2).

### 3.2 Generic HRQoL

Generic HRQoL was high, with a mean EQ‐5D VAS of 82 ± 13 and EQ‐5D index value of 0.94 ± 0.09 at inclusion (Table 1). No clinically relevant differences were found between the diagnostic modalities.

**TABLE 1:** EuroQol five dimensions (EQ‐5D) scores.

	Baseline		After 2–3 weeks		Change mean vs. baseline (95% CI)	After 6 months		Change mean vs. after 2–3 weeks (95% CI)
	Mean (SD)	n	Mean (SD)	n		Mean (SD)	n	
***Equation 5D index***									
**All patients**	0.94 (0.09)	528	0.95 (0.09)	455	0.00 (−0.01,0.01)	0.94 (0.09)	392	0.00 (−0.01,0.01)
**Patients that underwent MRI, MRGB and TRUSGB**	0.94 (0.09)	265	0.94 (0.09)	239	−0.01 (−0.02,0.01)	0.94 (0.1)	207	0.00 (−0.01,0.01)
**Patients that underwent MRI and TRUSGB**	0.94 (0.09)	263	0.96 (0.08)	216	0.01 (0.00,0.02)	0.95 (0.08)	185	−0.00 (−0.01,0.01)
**PCa detected**	0.94 (0.1)	276	0.94 (0.09)	252	−0.01 (−0.02,0.01)	0.94 (0.1)	217	0.00 (−0.01,0.02)
**No PCa detected**	0.94 (0.09)	251	0.96 (0.08)	203	0.01 (0.00,0.02)	0.95 (0.08)	175	−0.00 (−0.01,0.00)
**cisPCa detected**	0.93 (0.09)	117	0.94 (0.09)	106	0.01 (−0.01,0.03)	0.95 (0.09)	86	0.00 (−0.01,0.02)
**csPCa cancer detected**	0.95 (0.09)	160	0.93 (0.09)	146	−0.02 (−0.04,‐0.01)	0.94 (0.1)	128	0.01 (−0.01,0.02)
***Equation 5 D VAS***									
**All patients**	82 (13)	525	81 (11)	463	−1.2 (−1.3,‐0.1)	80 (13)	393	−0.7 (−1.7,0.4)
**Patients that underwent MRI, MRGB and TRUSGB**	81 (15)	263	81 (12)	242	−0.4 (−2.2,1.4)	79 (14)	209	−1.4 (−3.1,0.3)
**Patients that underwent MRI and TRUSGB**	83 (10)	262	81 (11)	221	−2.1 (−3.1,−1.0)	81 (11)	184	0.2 (−1.0,1.3)
**PCa detected**	81 (15)	277	80 (12.)	256	−1.0 (−2.7,0.8)	79 (14)	218	‐1.0 (−2.6,0.7)
**No PCa detected**	84 (10)	247	82 (10)	207	−1.5 (−2.6,‐0.4)	81 (11)	175	−0.3 (−1.4,0.9)
**cisPCa detected**	81 (14)	117	80 (12)	107	−1.9 (−4.5,0.8)	78 (13)	87	−0.6 (−3.1,2.0)
**csPCa cancer detected**	81 (16)	161	81 (13)	149	−0.5 (−2.8,1.9)	79 (14)	128	−1.5 (−3.8,0.7)

*Note:* Displayed results are mean scores (standard deviation).

Abbreviations: MRI = Magnetic Resonance Imaging, MRGB = targeted MR guided biopsy, TRUSGB = systematic transrectal ultrasound guided biopsies, VAS = visual analogue scale, used in Equation 5D. SD = standard deviation, PCa = prostate cancer, cisPCa = clinically insignificant PCa, csPCa = clinically significant PCa.

### 3.3 Cancer‐specific HRQoL

Figure 4A shows an overview of QLQ‐C30 scores. At baseline, results of all function scales were similar in patients who underwent mpMRI and TRUSGB, and patients who underwent mpMRI, TRUSGB and MRGB (Table S3). After 2–3 weeks and 6 months, scores of all function scales were lower in patients who underwent mpMRI, TRUSGB and MRGB compared to patients who underwent mpMRI and TRUSGB. The biggest differences were seen after 6 months in role function (95 ± 14 in patients that underwent mpMRI and TRUSGB, vs. 87 ± 22 in patients that underwent mpMRI, TRUSGB and MRGB) and emotional function (93 ± 12 vs. 88 ± 18, respectively). Regarding symptom scales, the largest differences were seen in fatigue. In patients that underwent mpMRI and TRUSGB, fatigue scores were 9.8 ± 15 at baseline, 12 ± 16 after 2–3 weeks and 9.8 ± 14 after 6 months, compared to 10 ± 16, 17 ± 20 and 18 ± 20 for patients that underwent both MRGB and TRUSGB, respectively. However, all differences did not meet the MICD thresholds.

**FIGURE 4A:**
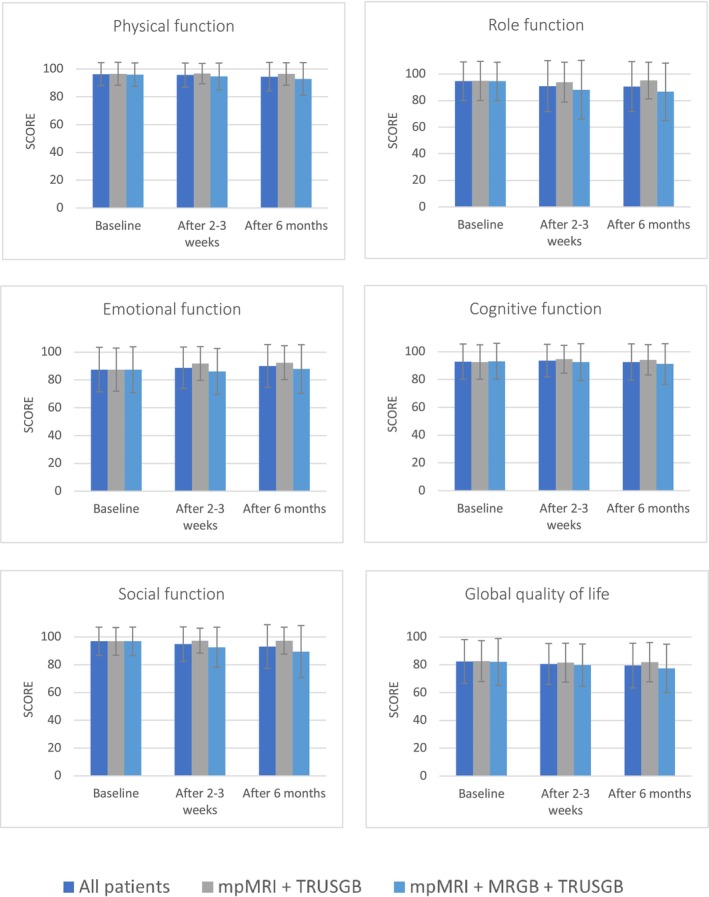
**QLQ‐C30 functioning scales according to diagnostic procedure. Scores range from 0 to 100; a higher score represents a better level of functioning**. *MRI = magnetic resonance imaging, MRGB = targeted MR guided biopsy, TRUSGB = systematic transrectal ultrasound guided biopsies.*

### 3.4 PCa‐specific HRQoL

Figure 4B shows an overview of QLQ‐PR25 scales. Clinically relevant differences were seen in incontinence aid and hormonal treatment‐related symptoms after 6 months, compared between patients that underwent mpMRI and TRUSGB and patients that underwent mpMRI, TRUSGB and MRGB (Table S3). In patients who underwent mpMRI and TRUSGB, scores after 6 months were 13 ± 13 for urinary symptoms, 10 ± 16 for incontinence aid, 3.3 ± 5.9 for hormonal treatment‐related symptoms and 38 ± 21 for sexual activity. In patients that underwent mpMRI, TRUSGB and MRGB, these scores were 20 ± 18, 18 ± 27, 7.9 ± 9.7 and 27 ± 20, respectively.

**FIGURE 4B:**
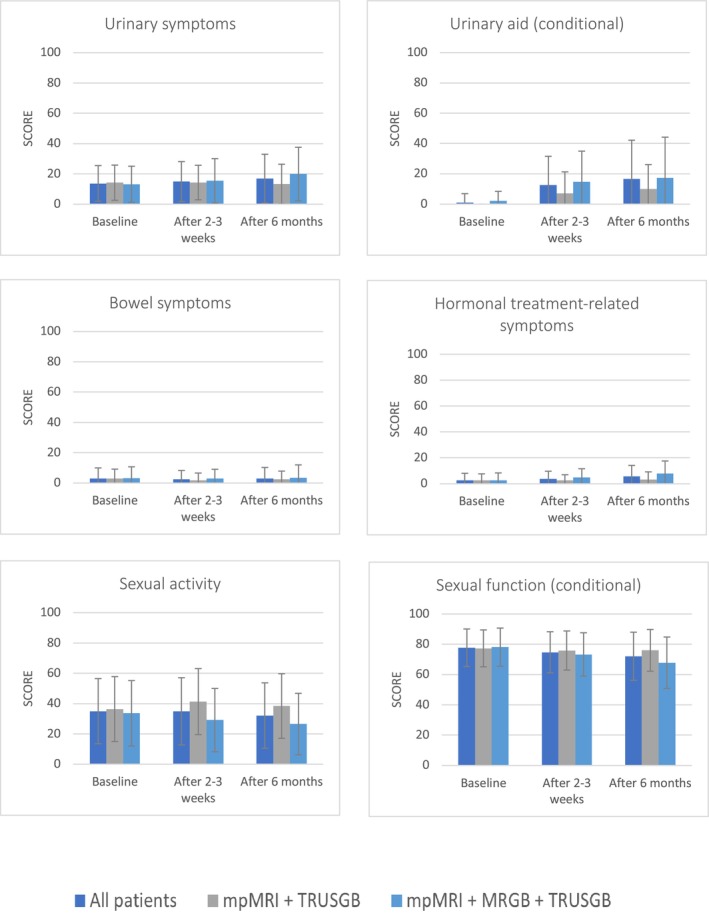
**QLQ‐CPR25 scales according to diagnostic procedure. Scores range from 0 to 100; a higher functional score reflects better function, while higher symptom scores reflect worsened symptoms.** *MRI = magnetic resonance imaging, MRGB = targeted MR‐guided biopsy, TRUSGB = systematic transrectal ultrasound‐guided biopsies.*

Overall, PCa was detected in 334 men (53%). 57% was csPCa (190/334) and 43% was cisPCa (144/334). The detection rate in patients that underwent mpMRI and TRUSGB was 24% (73/309), versus 82% (261/317) in patients that underwent mpMRI, TRUSGB and MRGB. Of the 334 men with PCa, 131 underwent radical prostatectomy (39%), 59 external radiotherapy (18%), 40 brachytherapy (12%), 51 hormonal therapy (15%), 4 hormonal therapy+chemotherapy (1%) and 88 active surveillance (26%).

A comparison of PCa patients and those without PCa showed lower scores of the PCa scale incontinence aid after 2–3 weeks and hormonal treatment‐related symptoms after 6 months in PCa patients (Table S4). Outcomes stratified to treatment types are shown in Table S5.

## 4 DISCUSSION

Our objective was to describe and compare anxiety and HRQoL in men who underwent mpMRI and TRUSGB and men who underwent mpMRI, MRGB and TRUSGB due to an elevated PSA.

Based on earlier studies, we expected lower anxiety values after mpMRI and MRGB, but we did not find any clinically relevant differences in anxiety scores in patients who underwent mpMRI and TRUSGB compared to patients who underwent mpMRI, MRGB and TRUSGB. We also did not find any clinically relevant differences in generic and cancer‐specific HRQoL after 2–3 weeks and 6 months.

Patients who underwent mpMRI, MRGB and TRUSGB had clinically relevant lower PCa‐specific HRQoL scores on incontinence aid and hormonal treatment‐related symptoms after 6 months compared to patients who underwent mpMRI and TRUSGB. This is probably influenced by a higher PCa detection rate.

Our results are not in agreement with Chesnut et al., who demonstrated higher anxiety scores in men who received targeted and systematic biopsies compared to systematic biopsies alone, although the differences they found were probably not clinically relevant.^6^ Shanker et al. measured the influence of MRI and TRUSGB on anxiety in men in an active surveillance protocol.^7^ This study showed significantly lower anxiety scores in patients who underwent MRI compared to TRUSGB. However, it could be questioned if this small difference is clinically relevant. A few studies investigated the influence of TRUSGB on anxiety and showed that anxiety increased during the diagnostic pathway and peaked just before patients received biopsy results.^18^, ^19^, ^20^ We could not confirm these results, as no peak in anxiety was seen after TRUSGB. As all our patients underwent (systematic and/or targeted) biopsies after mpMRI, this could have led to higher anxiety scores after mpMRI because of anticipation of discomfort or persistent insecurity. Therefore, our results could also reflect the substantial impact of a diagnostic PCa procedure.

Our results are in agreement with Kasivisvanathan et al and Shanker et al, who also found no decrease in generic HRQOL shortly after mpMRI and TRUSGB.^7^, ^8^

We showed lower PCa‐specific HRQoL scores on incontinence aid and hormonal treatment‐related symptoms during follow‐up in patients who underwent mpMRI, MRGB and TRUSGB. To our knowledge, no other studies have compared the influence of TRUSGB and MRGB on PCa‐related HRQoL. Some studies evaluated the impact of TRUSGB on components of HRQoL, like erectile or voiding function.^21^, ^22^, ^23^, ^24^ A review concluded that TRUSGB appears to be associated with short‐term exacerbation of urinary symptoms and temporary erectile dysfunction, although evidence is sparse.^25^ The difference in results after 6 months could be explained by the presence of PCa, as the presence of PCa varied from 24% in patients that underwent mpMRI and TRUSGB compared to 82% in patients that underwent mpMRI, MRGB and TRUSGB. Differences at this time point will probably reflect the influence of different types of PCa treatment.^26^

The major strength of this study is a large longitudinal analysis investigating anxiety and HRQoL in men suspected of PCa during their diagnostic pathway. Moreover, we measured HRQoL by multiple questionnaires focusing on different aspects of HRQoL and compared different diagnostic modalities. Some limitations should also be discussed. First, this was not a randomized controlled trial. All patients underwent mpMRI and TRUSGB, making the addition of MRGB in one arm the only difference between the pathways. This could have influenced the lack of difference between the pathways. Moreover, results could have been influenced by previous or subsequent examinations.

Furthermore, patients were not blinded to the results of the mpMRI.

Second, non‐response could have introduced bias. Response rates varied from 87% at baseline to 61% after 6 months. Researchers strive for a response rate above 80%, although there is no evidence regarding minimum valid response rates.^27^ Moreover, our results are comparable with average response rates of 75% at baseline and 67% at a follow‐up within one year found in a meta‐analysis of studies measuring patient‐reported outcome measures.^28^

Third, QLQ‐C30 and especially QLQ‐PR25 did not show good results on all psychometric properties.^29^ For the QLQ‐C30, some data were missing to enable a complete assessment. However, it showed good responsiveness, which we considered as a particularly important property characteristic, as effects of procedures and treatments had to be compared. For the QLQ‐PR25, several properties, like responsiveness and construct validity, were doubtful.^29^ This could lead to missing clinically important changes within subjects over time, and a lower correlation with the outcomes that were intended to measure. However, we chose QLQ‐C30 and QLQ‐PR25 as they are widely used and offer the possibility to combine general and PCa‐specific HRQoL.

We decided to use MICDs for the interpretation of results, as these represent changes considered worthwhile by a patient. This information is more useful than statistical significance, which does not necessarily imply clinical relevance. However, thresholds for MICDs were not available for all questionnaires used. Moreover, MICDs are initially developed to detect meaningful changes in individual scores. We also used it for the interpretation of mean group differences, as it is likely that meaningful changes on this level will be of a comparable size.

Fourth, confounding by indication could not be excluded. The biopsy scheme was based on PI‐RADS score, and treatment was influenced by biopsy outcomes.

Our hypothesis was that patients who underwent mpMRI and TRUSGB would show lower anxiety scores and better HRQoL scores compared to those who underwent mpMRI, MRGB and TRUSGB. However, this study did not demonstrate clinically relevant differences between the different diagnostic pathways. This means that all diagnostic PCa procedures can influence anxiety, even a reassuring mpMRI result. Urologists should be aware of this psychological impact, which should be discussed with patients in the context of shared decision‐making.

## 5 CONCLUSIONS

In men suspected of PCa, no differences were observed in anxiety levels or generic HRQoL scores across different diagnostic pathways. However, lower PCa‐specific HRQoL subscores were noted in patients who underwent mpMRI, MRGB and TRUSGB.

## AUTHOR CONTRIBUTIONS

Esther H.J. Hamoen had full access to all the data in the study and takes responsibility for the integrity of the data and the accuracy of the data analysis. Study concept and design: Hamoen, Israël, van der Leest, Rovers, Barentsz. Acquisition of data: van der Leest, Cornel, Israël, Hendriks, Veltman, van Oort, Barentsz. Analysis and interpretation of data: Hamoen, Israël, van der Leest, Hannink, Rovers, Barentsz. Drafting of the manuscript: Hamoen, Israël, Rovers. Critical revision of the manuscript for important intellectual content: Hamoen, Israël, van der Leest, Cornel, Klaver, Hendriks, Veltman, van Oort, Hannink, Witjes, Barentsz, Rovers. Statistical analysis: Hamoen, Rovers. Obtaining funding: Rovers, Barentsz. Supervision: Barentsz, Rovers.

## CONFLICT OF INTEREST STATEMENT

The authors declare no conflicts of interest.

## Supporting information


**Table S1.** **Overview of used HRQoL questionnaires and mean important clinical differences (MICD).** Numbers in italic are distribution‐based values at 0.5 standard deviation of our baseline results.

**Table S2.** State‐Trait Anxiety Inventory (STAI‐6) scores.

**Table S3.** Mean scores for general and disease‐specific health‐related quality of life in subgroups that underwent mpMRI and TRUSGB, and mpMRI in combination with MRGB and TRUSGB.

**Table S4.** Mean scores for general and disease‐specific health‐related quality of life, specified for men with PCa detected and men without Pca.

**Table S5.** Mean scores for general and disease‐specific health‐related quality of life, specified for PCa treatments.
