# Supplementary material for: Impact of multiparametric MRI and prostate biopsies on anxiety and quality of life in men with suspected prostate cancer
Source: BJUI Compass. 2025 Oct 17;6(10):e70087. doi: 10.1002/bco2.70087 (PMC12531450; doi:10.1002/bco2.70087)
Supplement: Supplementary file 1 — Table S1. Overview of used HRQoL questionnaires and mean important clinical differences (MICD). Numbers in italic are distribution‐based values at 0.5 standard deviation of our baseline results. [file BCO2-6-e70087-s001.docx]

**Supplemental Table 1. Overview of used HRQoL questionnaires and mean important clinical differences (MICD).** Numbers in italic are distribution-based values at 0.5 standard deviation of our baseline results.

|  | **No. items** | **Scale** | **MICD** |
| --- | --- | --- | --- |
| **EQ5D** |  |  |  |
| EQ5D index value | 5 | 0-1 | *0.044* |
| EQ5D VAS | 1 | 0-100 | 7-11 |
| **EORTC QLQ-C30** |  |  |  |
| Global QoL | 1 | 0-100 | 9.2 |
| Physical function | 5 | 0-100 | 7.9 |
| Role function | 2 | 0-100 | 9.3 |
| Emotional function | 4 | 0-100 | 9.3 |
| Cognitive function | 2 | 0-100 | 8.4 |
| Social function | 2 | 0-100 | 7.8 |
| Fatigue | 3 | 0-100 | 9.3 |
| Nausea/vomiting | 2 | 0-100 | 3.3 |
| Pain | 1 | 0-100 | 8.9 |
| Dyspnea | 1 | 0-100 | 10.2 |
| Insomnia | 1 | 0-100 | 12.5 |
| Appetite loss | 1 | 0-100 | 5.8 |
| Constipation | 1 | 0-100 | 9.2 |
| Diarrhea | 1 | 0-100 | 7.5 |
| Financial difficulties | 1 | 0-100 | *3.5* |
| **EORTC QLQ-PR25** |  |  |  |
| Urinary symptoms | 8 | 0-100 | *5.9* |
| Use of incontinence aid | 1 | 0-100 | *3.0* |
| Bowel symptoms | 4 | 0-100 | *3.5* |
| Hormonal therapy-related symptoms | 6 | 0-100 | *2.7* |
| Sexual activity | 2 | 0-100 | *10.8* |
| Sexual functioning | 4 | 0-100 | *6.2* |
| **STAI-6** | 6 | 20-80 | *4.8* |
|  |  |  |  |

Abbreviatons: EQ5D = EuroQol five dimensions, VAS = visual analogue scale, QLQ-C30 = European Organisation for Research and Treatment of Cancer Quality of Life Questionnaire Core 30, QLQ-PR25 = European Organisation for Research and Treatment of Cancer Quality of Life Questionnaire Prostate Cancer Module
