# Supplementary material for: Impact of multiparametric MRI and prostate biopsies on anxiety and quality of life in men with suspected prostate cancer
Source: BJUI Compass. 2025 Oct 17;6(10):e70087. doi: 10.1002/bco2.70087 (PMC12531450; doi:10.1002/bco2.70087)
Supplement: Supplementary file 2 — Table S2. State‐Trait Anxiety Inventory (STAI‐6) scores. [file BCO2-6-e70087-s003.docx]

**Supplemental Table 2. State-Trait Anxiety Inventory (STAI-6) scores.**

|  | **Baseline** |  | **After MRI** |  | **Change mean vs. baseline (95% CI)** | **After TRUSGB** |  | **Change mean vs. after MRI (95% CI)** | **After 2-3 weeks** |  | **Change mean vs. after TRUSGB (95% CI)** | **After 6 months** |  | **Change mean vs. after 2-3 weeks (95% CI)** |
| --- | --- | --- | --- | --- | --- | --- | --- | --- | --- | --- | --- | --- | --- | --- |
|  | **Mean (SD)** | **n** | **Mean (SD)** | **n** |  | **Mean (SD)** | **n** |  |  |  |  | **Mean (SD)** | **n** |  |
| **All patients** | 35.7 (9.6) | 525 | 39.5 (9.8) | 523 | 3.8 (2.9,4.6) | 37.3 (9.7) | 509 | -2.1 (-2.8,-1.4) | 33.0 (9.3) | 456 | -4.3 (-5.3,-3.2) | 32.4 (9.0) | 384 | -0.2 (-1.0,0.5) |
| **Patients that underwent MRI, MRGB and TRUSGB** | 36.0 (9.9) | 262 | 40.3 (9.8) | 269 | 4.2 (3.0-5.4) | 38.1 (9.9) | 259 | -2.1 (-3.0,--1.1) | 33.9 (9.5) | 238 | -3.8 (-5.1,-2.5) | 33.1 (9.2) | 204 | -0.8 (-1.8,0.3) |
| **Patients that underwent MRI and TRUSGB** | 35.4 (9.4) | 263 | 38.7 (9.8) | 254 | 3.3 (2.0-4.9) | 36.4 (9.5) | 250 | -2.2 (-3.3,-1.0) | 32.0 (9.0) | 218 | -4.7 (-6.3,-3.1) | 31.6 (8.7) | 180 | 0.4 (-0.7,1.4) |
| **PCa detected** | 36.0 (10.0) | 274 | 40.0 (9.6) | 282 | 4.0 (2.7-5.2) | 38.0 (9.9) | 275 | -1.9 (-2.9,-0.9) | 34.5 (9.8) | 250 | -3.4 (-4.7,-2.0) | 32.7 (9.3) | 212 | -1.5 (-2.6,-0.5) |
| **No Pca detected** | 35.5 (9.2) | 250 | 39.0 (10.0) | 241 | 3.5 (2.3-4.7) | 36.4 (9.4) | 234 | -2.4 (-3.5,-1.3) | 31.2 (8.4) | 206 | -5.3 (- 6.9,-3.7) | 32.1 (8.5) | 172 | 1.3 (0.3-2.4) |
| **cisPCa detected** | 36.3 (9.4) | 117 | 40.4 (8.9) | 120 | 4.0 (2.3-5.7) | 38.1 (9.0) | 117 | -2.1 (-3.6,-0.7) | 34.6 (9.5) | 105 | -3.6 (-5.6,-1.6) | 32.4 (8.9) | 84 | -1.3 (-3.0,0.4) |
| **csPCa detected** | 36.0 (10.8) | 159 | 39.8 (10.5) | 161 | 3.8 (2.0-5.5) | 38.1 (10.8) | 157 | -1.7 (-3.1,-1.7) | 34.2 (10.0) | 145 | -3.6 (-5.4,-1.8) | 32.8 (9.8) | 125 | -1.6 (-2.9,-0.2) |

Displayed results are mean scores (standard deviation).

*Abbreviations: MRI=Magnetic Resonance Imaging, MRGB=targeted MR guided biopsy, TRUSGB=systematic transrectal ultrasound guided-biopsies, SD = standard deviation, PCa = prostate cancer, cisPCa = clinically insignificant PCa, csPCa = clinically significant PCa*
