# Supplementary material for: Impact of multiparametric MRI and prostate biopsies on anxiety and quality of life in men with suspected prostate cancer
Source: BJUI Compass. 2025 Oct 17;6(10):e70087. doi: 10.1002/bco2.70087 (PMC12531450; doi:10.1002/bco2.70087)
Supplement: Supplementary file 3 — Table S3. Mean scores for general and disease‐specific health‐related quality of life in subgroups that underwent mpMRI and TRUSGB, and mpMRI in combination with MRGB and TRUSGB. [file BCO2-6-e70087-s005.docx]

**Supplemental Table 3. Mean scores for general and disease-specific health-related quality of life in subgroups that underwent mpMRI and TRUSGB, and mpMRI in combination with MRGB and TRUSGB**

*Abbreviations: MRI=Magnetic Resonance Imaging, MRGB=targeted MR guided biopsy, TRUSGB=systematic transrectal ultrasound guided-biopsies, QLQ-C30 = European Organisation for Research and Treatment of Cancer Quality of Life Questionnaire Core 30, QLQ-PR25 = European Organisation for Research and Treatment of Cancer Quality of Life Questionnaire Prostate Cancer Module, SD = standard deviation, CI = confidence interval*

|  | **Baseline** |  |  |  |  | **After 2-3 weeks** |  |  |  |  | **After 6 months** |  |  |  |  |
| --- | --- | --- | --- | --- | --- | --- | --- | --- | --- | --- | --- | --- | --- | --- | --- |
|  | MRI and TRUSGB |  | MRI, MRGB and TRUSGB |  | Difference between groups | MRI and TRUSGB |  | MRI, MRGB and TRUSGB |  | Difference between groups | MRI and TRUSGB |  | MRI, MRGB and TRUSGB |  | Difference between groups |
| **QLQ-C30 functioning scale** | **Mean (SD)** | **n** | **Mean (SD)** | **n** | **Mean (95% CI)** | **Mean (SD)** | **n** | **Mean (SD)** | **n** | **Mean (95% CI)** | **Mean (SD)** | **n** | **Mean (SD)** | **n** | **Mean (95% CI)** |
| **Global quality of life** | 82.7 (14.7) | 267 | 82.1 (16.8) | 277 | 0.6 (-2.0,3.3) | 81.6 (14.0) | 217 | 79.8 (15.2) | 240 | 1.9 (-0.8,4.5) | 81.9 (14.1) | 190 | 77.5 (17.4) | 220 | 4.5 (1.4,7.5) |
| **Physical function** | 96.6 (8.3) | 268 | 96.0 (8.4) | 277 | 0.6 (-0.8,2.0) | 96.7 (7.3) | 221 | 94.6 (9.7) | 241 | 2.2 (0.6,3.7) | 96.4 (8.1) | 189 | 92.9 (11.7) | 222 | 3.5 (1.5,5.4) |
| **Role function** | 94.8 (14.7) | 269 | 94.5 (14.4) | 278 | 0.3 (-2.1,2.8) | 93.9 (15.0) | 218 | 88.2 (22.1) | 238 | 5.6 (2.2,9.1) | 95.1 (13.8) | 190 | 86.6 (21.6) | 219 | 8.5 (5.0,12.0) |
| **Emotional function** | 87.5 (15.5) | 269 | 87.4 (16.5) | 277 | 0.1 (-2.6,2.8) | 91.9 (12.1) | 220 | 86.1 (16.6) | 241 | 5.8 (3.1,8.4) | 92.5 (12.2) | 190 | 87.9 (17.5) | 219 | 4.6 (1.7,7.5) |
| **Cognitive function** | 92.6 (12.4) | 269 | 93.1 (13.0) | 277 | -0.6 (-2.7,1.6) | 94.6 (10.0) | 220 | 92.6 (13.2) | 241 | 2.0 (-0.1,4.1) | 94.2 (10.9) | 190 | 91.2 (14.6) | 219 | 3.0 (0.6,5.5) |
| **Social function** | 96.8 (10.0) | 269 | 96.8 (10.3) | 277 | 0.0 (-1.7,1.7) | 97.3 (9.0) | 220 | 92.6 (14.4) | 241 | 4.7 (2.5,6.9) | 97.3 (9.7) | 190 | 89.4 (18.8) | 219 | 7.9 (5.0,10.7) |
| **QLQ-C30 symptom scales** |  |  |  |  |  |  |  |  |  |  |  |  |  |  |  |
| **Fatigue** | 9.8 (15.3) | 269 | 10.3 (15.9) | 277 | -0.5 (-3.1,2.1) | 11.9 (15.6) | 218 | 16.6 (20.0) | 238 | -4.8 (-8.1,-1.5) | 9.8 (14.4) | 190 | 18.0 (20.1) | 219 | -8.2 (-11.6,-4.8) |
| **Nausea and vomiting** | 0.7 (4.1) | 269 | 1.4 (6.9) | 277 | -0.7 (-1.7,0.3) | 1.1 (5.6) | 221 | 1.4 (5.9) | 242 | -0.3 (-1.4,0.7) | 0.6 (3.6) | 190 | 0.9 (4.1) | 220 | -0.3 (-1.1,0.5) |
| **Pain** | 5.6 (14.3) | 269 | 7.0 (15.0) | 278 | -1.3 (-3.8,1.2) | 6.2 (13.2) | 221 | 7.6 (16.0) | 242 | -1.5 (4.2,1.2) | 5.3 (12.9) | 190 | 8.4 (16.9) | 220 | -3.1 (-6.0,-0.2) |
| **Dyspnea** | 5.0 (12.6) | 269 | 5.5 (14.2) | 278 | -0.6 (-2.8,1.7) | 5.4 (12.7) | 217 | 5.7 (16.2) | 238 | -0.4 (-3.1,2.3) | 5.3 (13.1) | 190 | 6.1 (15.1) | 219 | -0.8 (-3.6,1.9) |
| **Insomnia** | 11.8 (21.9) | 269 | 10.9 (19.5) | 278 | 0.9 (-2.6,4.3) | 10.7 (19.4) | 218 | 14.4 (23.4) | 238 | -3.7 (-7.7,0.2) | 9.1 (17.8) | 190 | 15.1 (23.9) | 219 | -5.9 (-10.0,-1.9) |
| **Appetite loss** | 1.2 (7.5) | 269 | 1.4 (7.4) | 277 | -0.2 (-1.5,1.0) | 1.2 (7.7) | 218 | 2.2 (10.4) | 238 | -1.0 (-2.7,0.7) | 0.4 (3.4) | 190 | 3.2 (13.4) | 219 | -2.8 (-4.7,-1.0) |
| **Constipation** | 3.0 (10.4) | 269 | 4.0 (12.6) | 276 | -1.0 (-2.9,0.9) | 4.1 (12.7) | 220 | 4.8 (12.9) | 241 | -0.8 (-3.1,1.6) | 2.5 (10.0) | 190 | 4.3 (13.2) | 219 | -1.8 (-4.1,0.5) |
| **Diarrhea** | 3.5 (10.6) | 268 | 4.6 (13.1) | 277 | -1.1 (-3.1,0.9) | 4.1 (13.1) | 220 | 3.6 (11.6) | 241 | 0.5 (-1.8,2.8) | 2.6 (9.6) | 190 | 4.6 (12.8) | 218 | -2.0 (-4.1,0.2) |
| **Financial difficulties** | 0.6 (5.4) | 269 | 1.3 (8.2) | 276 | -0.7 (-1.9,0.5) | 1.2 (7.7) | 219 | 4.0 (13.5) | 241 | -2.8 (-4.8,0.8) | 0.9 (6.4) | 190 | 4.4 (14.1) | 219 | -3.5 (-5.6,-1.5) |
| **QLQ-PR25 scales** |  |  |  |  |  |  |  |  |  |  |  |  |  |  |  |
| **Urinary symptoms** | 14.2 (11.6) | 266 | 13.2 (11.9) | 276 | 1.1 (-0.9,3.1) | 14.3 (11.4) | 221 | 15.4 (14.7) | 241 | -1.1 (-3.5,1.3) | 13.3 (13.1) | 190 | 19.9 (17.7) | 219 | -6.6 (-9.6,-3.6) |
| **Incontinence aid (conditional)** | 0.0 (0.0) | 16 | 2.1 (6.3) | 16 | -2.1 (-6.5,2.4) | 7.1 (14.2) | 14 | 14.8 (20.2) | 36 | -7.7 (-18.0,2.6) | 10.0 (16.1) | 10 | 17.4 (26.8) | 67 | -7.4 (-24.8,10.0) |
| **Bowel symptoms** | 2.9 (6.3) | 257 | 3.1 (7.6) | 273 | -0.2 (-1.4,1.0) | 1.8 (4.8) | 216 | 2.9 (6.2) | 237 | -1.1 (-2.1,-0.1) | 2.5 (5.4) | 185 | 3.5 (8.5) | 215 | -1.0 (-2.4,0.4) |
| **Hormonal treatment-related symptoms** | 2.7 (5.0) | 264 | 2.8 (5.6) | 275 | -0.1 (-1.0,0.8) | 2.8 (4.2) | 216 | 4.9 (6.7) | 239 | -2.1 (-3.1,-1.1) | 3.3 (5.9) | 189 | 7.9 (9.7) | 217 | -4.6 (-6.2,-3.1) |
| **Sexual activity** | 36.4 (21.4) | 262 | 33.6 (21.6) | 272 | 2.7 (-0.9,6.4) | 41.3 (21.8) | 216 | 29.1 (20.9) | 236 | 12.2 (8.2,16.1) | 38.4 (21.3) | 189 | 26.5 (20.3) | 216 | 11.8 (7.7,15.9) |
| **Sexual function (conditional)** | 77.3 (12.2) | 222 | 78.1 (12.6) | 210 | -0.8 (-3.2,1.5) | 75.9 (12.9) | 185 | 73.3 (14.3) | 165 | 2.6 (-0.2,5.5) | 76.0 (13.8) | 159 | 67.8 (17.0) | 143 | 8.1 (4.6,11.7)( |
