# Supplementary material for: Impact of multiparametric MRI and prostate biopsies on anxiety and quality of life in men with suspected prostate cancer
Source: BJUI Compass. 2025 Oct 17;6(10):e70087. doi: 10.1002/bco2.70087 (PMC12531450; doi:10.1002/bco2.70087)
Supplement: Supplementary file 4 — Table S4. Mean scores for general and disease‐specific health‐related quality of life, specified for men with PCa detected and men without Pca. [file BCO2-6-e70087-s002.docx]

**Supplemental Table 4. Mean scores for general and disease-specific health-related quality of life, specified for men with PCa detected and men without PCa**

*Abbreviations: PCa = prostate cancer, QLQ-C30 = European Organisation for Research and Treatment of Cancer Quality of Life Questionnaire Core 30, QLQ-PR25 = European Organisation for Research and Treatment of Cancer Quality of Life Questionnaire Prostate Cancer Module, SD = standard deviation, CI = confidence interval*

|  | **Baseline** |  |  |  |  | **After 2-3 weeks** |  |  |  |  | **After 6 months** |  |  |  |  |
| --- | --- | --- | --- | --- | --- | --- | --- | --- | --- | --- | --- | --- | --- | --- | --- |
|  | **PCa detected** |  | **PCa not detected** |  | Difference between groups | **PCa detected** |  | **PCa not detected** |  | Difference between groups | **PCa detected** |  | **PCa not detected** |  | Difference between groups |
| **QLQ-C30 functioning scale** | **Mean (SD)** | **n** | **Mean (SD)** | **n** | **Mean (95% CI)** | **Mean (SD)** | **n** | **Mean (SD)** | **n** | **Mean (95% CI)** | **Mean (SD)** | **n** | **Mean (SD)** | **n** | **Mean (95% CI)** |
| **Global quality of life** | 81.7 (16.8) | 289 | 83.0 (14.5) | 254 | 1.1 (-1.5,3.8) | 79.3 (15.1) | 252 | 82.4 (14.0) | 205 | 3.1 (0.4,5.8) | 77.7 (17.2) | 227 | 81.8 (14.1) | 183 | 4.2 (1.1,7.2) |
| **Physical function** | 95.7 (9.0) | 289 | 97.0 (7.5) | 255 | 1.4 (-0.0,2.7) | 94.7 (9.5) | 256 | 96.7 (7.6) | 206 | 2.0 (0.4,3.5) | 92.9 (11.7) | 229 | 96.6 (7.9) | 182 | 3.6 (1.7,5.5) |
| **Role function** | 94.3 (14.7) | 290 | 95.0 (14.5) | 256 | 0.7 (-1.8,3.1) | 88.4 (21.8) | 251 | 94.0 (14.8) | 205 | 5.5 (2.1,8.4) | 87.2 (21.5) | 226 | 94.6 (13.9) | 183 | 7.4 (3.9,10.8) |
| **Emotional function** | 87.9 (16.3) | 289 | 87.0 (15.7) | 256 | -0.9 (-3.6,1.8) | 86.3 (16.2) | 255 | 92.0 (12.4) | 206 | 5.7 (3.1,8.3) | 88.5 (16.6) | 226 | 92.0 (13.6) | 183 | 3.5 (0.6,6.4) |
| **Cognitive function** | 93.0 (13.0) | 289 | 92.6 (12.3) | 256 | -0.4 (-2.5,1.8) | 92.7 (12.8) | 255 | 94.7 (10.3) | 206 | 2.0 (-0.1,4.1) | 91.6 (13.8) | 226 | 93.8 (12.0) | 183 | 2.2 (-0.3,4.7) |
| **Social function** | 96.7 (10.2) | 289 | 96.9 (10.1) | 256 | 0.2 (-1.5,1.9) | 92.5 (14.4) | 255 | 97.7 (8.5) | 206 | 5.1 (3.0,7.2) | 89.9 (18.7) | 226 | 97.0 (9.8) | 183 | 7.1 (4.3,9.9) |
| **QLQ-C30 symptom scales** |  |  |  |  |  |  |  |  |  |  |  |  |  |  |  |
| **Fatigue** | 9.9 (15.8) | 289 | 10.3 (15.5) | 254 | 0.4 (-2.2,3.0) | 16.6 (19.7) | 251 | 11.6 (15.8) | 205 | -5.0 (-8.3,-1.8) | 17.4 (19.7) | 226 | 10.3 (15.3) | 183 | -7.0 (-10.4,-3.6) |
| **Nausea and vomiting** | 1.2 (6.6) | 289 | 0.8 (4.5) | 255 | -0.4 (1.3,6.0) | 1.4 (5.9) | 256 | 1.0 (5.6) | 207 | -0.5 (-1.5,0.6) | 0.8 (3.6) | 227 | 0.7 (4.2) | 183 | -0.1 (0.8,0.7) |
| **Pain** | 7.4 (16.4) | 290 | 5.1 (12.4) | 256 | -2.2 (-4.6,0.2) | 7.4 (15.6) | 256 | 6.4 (13.6) | 207 | -1.1 (-3.8,1.6) | 7.8 (16.0) | 227 | 5.9 (14.2) | 183 | -1.9 (-4.8,1.1) |
| **Dyspnea** | 5.5 (14.1) | 290 | 4.9 (12.6) | 256 | -0.6 (-2.8,1.7) | 5.9 (15.8) | 250 | 5.2 (13.0) | 205 | -0.7 (-3.4,2.0) | 6.2 (15.4) | 226 | 5.1 (12.5) | 183 | -1.1 (3.9,1.7) |
| **Insomnia** | 9.8 (18.8) | 290 | 13.2 (22.6) | 256 | 3.4 (-0.1,6.9) | 14.7 (23.1) | 251 | 10.1 (19.4) | 205 | -4.7 (-8.6,-0.7) | 13.1 (22.2) | 226 | 11.3 (20.5) | 183 | -1.8 (-6.0,2.4) |
| **Appetite loss** | 1.5 (8.0) | 289 | 1.2 (6.8) | 256 | -0.3 (-1.6,0.9) | 2.1 (10.1) | 251 | 1.3 (8.0) | 205 | -0.8 (-2.5,0.9) | 2.8 (12.8) | 226 | 0.7 (4.9) | 183 | -2.1 (-3.9,-0.2) |
| **Constipation** | 4.2 (12.7) | 289 | 2.7 (10.1) | 254 | -1.4 (-3.3,0.5) | 5.0 (13.9) | 255 | 3.9 (11.2) | 206 | -1.1 (-3.4,1.3) | 4.7 (14.3) | 226 | 1.8 (7.6) | 183 | -2.9 (-5.1,-0.7) |
| **Diarrhea** | 4.3 (12.5) | 288 | 3.8 (11.4) | 255 | -0.5 (-2.5,1.5) | 4.3 (12.7) | 255 | 3.2 (11.9) | 206 | -1.1 (-3.3,1.2) | 4.6 (12.7) | 225 | 2.6 (9.5) | 183 | -2.0 (-4.2,0.1) |
| **Financial difficulties** | 1.4 (8.2) | 289 | 0.5 (5.1) | 256 | -0.9 (-2.0,0.3) | 4.2 (13.9) | 255 | 0.8 (6.1) | 205 | -3.4 (-5.3,-1.5) | 4.3 (13.9) | 226 | 0.9 (6.5) | 183 | -3.4 (-5.4,-1.3) |
| **QLQ-PR25 scales** |  |  |  |  |  |  |  |  |  |  |  |  |  |  |  |
| **Urinary symptoms** | 13.2 (12.1) | 287 | 14.3 (11.4) | 254 | 1.1 (-0.8,3.1) | 16.5 (14.9) | 255 | 12.8 (10.5) | 207 | -3.7 (-6.0,-1.3) | 20.7 (17.8) | 226 | 12.1 (12.1) | 183 | -8.6 (-11.5,-5.7) |
| **Incontinence aid (conditional)** | 0.0 (0.0) | 18 | 2.6 (9.2) | 13 | 2.6 (-3.0,8.2) | 15.4 (19.9) | 41 | 0.0 (0.0) | 9 | -15.4 (-21.7,-9.2) | 17.9 (26.6) | 69 | 4.2 (11.8) | 8 | -13.7 (-24.8,-2.6) |
| **Bowel symptoms** | 3.1 (7.5) | 283 | 2.9 (5.3) | 246 | -0.2 (-1.4,1.0) | 3.1 (6.4) | 250 | 1.5 (4.1) | 203 | -1.7 (-2.6,-0.7) | 3.7 (8.7) | 220 | 2.2 (5.0) | 180 | -1.5 (-2.8,-0.1) |
| **Hormonal treatment-related symptoms** | 2.7 (5.4) | 286 | 2.9 (5.3) | 252 | 0.3 (-0.6,1.2) | 5.0 (6.8) | 251 | 2.5 (3.8) | 204 | -2.4 (-3.4,-1.4) | 7.9 (9.8) | 223 | 3.1 (5.5) | 183 | -4.8 (-6.3,-3.3) |
| **Sexual activity** | 34.0 (20.9) | 283 | 36.1 (22.2) | 250 | 2.0 (-1.6,5.7) | 30.4 (22.3) | 248 | 40.4 (20.8) | 204 | 9.9 (5.9,13.9) | 26.7 (20.3) | 222 | 38.5 (21.4) | 183 | 11.8 (7.7,15.9) |
| **Sexual function (conditional)** | 77.5 (13.6) | 226 | 77.9 (11.0) | 206 | 0.4 (-2.0,2.7) | 72.7 (15.0) | 176 | 76.7 (11.6) | 174 | 4.1 (1.3,6.9) | 68.0 (17.7) | 154 | 76.4 (12.5) | 148 | 8.4 (4.9,11.8) |
