# Supplementary material for: Impact of multiparametric MRI and prostate biopsies on anxiety and quality of life in men with suspected prostate cancer
Source: BJUI Compass. 2025 Oct 17;6(10):e70087. doi: 10.1002/bco2.70087 (PMC12531450; doi:10.1002/bco2.70087)
Supplement: Supplementary file 5 — Table S5. Mean scores for general and disease‐specific health‐related quality of life, specified for PCa treatments. [file BCO2-6-e70087-s004.docx]

**Supplemental Table 5. Mean scores for general and disease-specific health-related quality of life, specified for PCa treatments**

*Abbreviations: PCa = prostate cancer, cisPCa = clinically insignificant PCa, csPCa = clinically significant PCa, QLQ-C30 = European Organisation for Research and Treatment of Cancer Quality of Life Questionnaire Core 30, QLQ-PR25 = European Organisation for Research and Treatment of Cancer Quality of Life Questionnaire Prostate Cancer Module, SD = standard deviation, RP = radical prostatectomy, EBRT = external beam radiotherapy, BT = brachytherapy, HT = hormonal therapy*

|  | **Baseline** |  |  |  |  |  |  |  | **After 2-3 weeks** |  |  |  |  |  |  |  |
| --- | --- | --- | --- | --- | --- | --- | --- | --- | --- | --- | --- | --- | --- | --- | --- | --- |
|  | **RP** |  | **EBRT** |  | **BT** |  | **HT** |  | **RP** |  | **EBRT** |  | **BT** |  | **HT** |  |
| **QLQ-C30 functioning scale** | **Mean (SD)** | **n** | **Mean (SD)** | **n** | **Mean (SD)** | **n** | **Mean (SD)** | **n** | **Mean (SD)** | **n** | **Mean (SD)** | **n** | **Mean (SD)** | **n** | **Mean (SD)** | **n** |
| **Global quality of life** | 83.3 (17.5) | 108 | 79.6 (17.0) | 56 | 82.4 (16.5) | 38 | 80.0 (17.3) | 48 | 81.1 (14.0) | 94 | 76.0 (15.8) | 48 | 78.3 (17.6) | 35 | 77.6 (16.7) | 42 |
| **Physical function** | 96.6 (7.5) | 109 | 93.0 (12.4) | 54 | 95.6 (9.6) | 38 | 93.3 (11.7) | 48 | 95.0 (10.1) | 95 | 92.5 (9.7) | 49 | 95.0 (8.9) | 35 | 93.0 (10.4) | 42 |
| **Role function** | 96.5 (12.2) | 109 | 92.4 (17.5) | 55 | 90.4 (19.2) | 38 | 90.6 (15.8) | 48 | 86.0 (25.6) | 94 | 82.3 (26.0) | 48 | 92.6 (14.9) | 34 | 83.7 (25.6) | 42 |
| **Emotional function** | 86.5 (17.5) | 108 | 88.2 (16.0) | 56 | 87.7 (20.8) | 38 | 91.0 (15.8) | 48 | 84.2 (18.2) | 94 | 85.5 (14.8) | 49 | 85.5 (19.3) | 35 | 87.3 (12.1) | 42 |
| **Cognitive function** | 93.7 (13.8) | 108 | 93.2 (10.9) | 56 | 89.9 (16.2) | 38 | 93.8 (10.7) | 48 | 92.7 (12.4) | 94 | 93.5 (13.1) | 49 | 91.4 (14.8) | 35 | 93.3 (11.7) | 42 |
| **Social function** | 96.9 (10.0) | 108 | 96.4 (10.9) | 56 | 94.7 (11.7) | 38 | 96.9 (10.7) | 48 | 90.6 (16.5) | 94 | 91.2 (14.5) | 49 | 91.9 (14.2) | 35 | 93.7 (12.7) | 42 |
| **QLQ-C30 symptom scales** |  |  |  |  |  |  |  |  |  |  |  |  |  |  |  |  |
| **Fatigue** | 9.2 (16.4) | 108 | 10.3 (15.1) | 55 | 8.2 (16.1) | 38 | 9.5 (14.7) | 48 | 14.8 (19.0) | 94 | 23.5 (22.3) | 48 | 15.0 (20.1) | 34 | 19.6 (22.7) | 42 |
| **Nausea and vomiting** | 2.2 (9.7) | 108 | 0.6 (3.1) | 56 | 0.3 (2.7) | 38 | 0.3 (2.4) | 48 | 1.4 (5.2) | 95 | 3.1 (10.0) | 49 | 1.0 (3.9) | 35 | 2.4 (9.4) | 42 |
| **Pain** | 5.4 (15.4) | 109 | 9.2 (18.0) | 56 | 8.8 (17.2) | 38 | 8.7 (18.5) | 48 | 7.4 (16.6) | 95 | 9.5 (16.7) | 49 | 4.8 (11.1) | 35 | 10.3 (17.2) | 42 |
| **Dyspnea** | 4.3 (12.1) | 109 | 7.3 (16.6) | 55 | 6.1 (15.2) | 38 | 7.6 (17.2) | 48 | 5.3 (15.7) | 94 | 9.0 (21.5) | 48 | 2.9 (9.6) | 34 | 8.7 (19.6) | 42 |
| **Insomnia** | 9.8 (18.9) | 109 | 11.5 (21.5) | 55 | 9.6 (20.4) | 38 | 11.1 (18.6) | 48 | 14.2 (22.1) | 94 | 25.7 (30.9) | 48 | 11.8 (21.5) | 34 | 25.4 (31.1) | 42 |
| **Appetite loss** | 1.9 (8.9) | 108 | 1.8 (7.6) | 55 | 0.9 (5.4) | 38 | 1.4 (6.7) | 48 | 3.2 (13.0) | 94 | 2.8 (11.6) | 48 | 1.0 (5.7) | 34 | 0.8 (5.1) | 42 |
| **Constipation** | 4.0 (12.7) | 108 | 6.5 (17.3) | 56 | 1.8 (7.5) | 38 | 5.6 (17.3) | 48 | 5.3 (14.9) | 94 | 3.4 (12.3) | 49 | 2.9 (9.5) | 35 | 3.2 (9.9) | 42 |
| **Diarrhea** | 5.9 (15.7) | 108 | 3.6 (10.4) | 56 | 1.8 (7.5) | 38 | 3.5 (10.3) | 48 | 3.9 (13.7) | 94 | 4.8 (11.8) | 49 | 2.9 (9.5) | 35 | 4.0 (10.9) | 42 |
| **Financial difficulties** | 0.9 (5.5) | 108 | 2.4 (14.0) | 56 | 2.6 (9.1) | 38 | 0.7 (4.8) | 48 | 3.2 (11.0) | 94 | 6.8 (19.2) | 49 | 7.6 (16.3) | 35 | 5.6 (14.6) | 42 |
| **QLQ-PR25 scales** |  |  |  |  |  |  |  |  |  |  |  |  |  |  |  |  |
| **Urinary symptoms** | 14.5 (12.3) | 108 | 12.6 (11.7) | 56 | 7.6 (9.6) | 38 | 11.8 (10.3) | 48 | 19.4 (15.7) | 94 | 16.6 (16.8) | 49 | 14.5 (15.4) | 35 | 13.9 (10.6) | 42 |
| **Incontinence aid (conditional)** | 0.0 (0.0) | 6 | 0.0 (0.0) | 5 | 0.0 (0.0) | 2 | 0.0 (0.0) | 5 | 19.2 (20.5) | 33 | 0.0 (0.0) | 5 | N.A. | 0 | 0.0 (0.0) | 3 |
| **Bowel symptoms** | 3.4 (6.5) | 106 | 3.0 (6.7) | 55 | 1.5 (3.3) | 38 | 2.5 (6.0) | 47 | 2.9 (5.4) | 93 | 3.8 (5.9) | 48 | 2.9 (10.2) | 34 | 2.8 (4.8) | 42 |
| **Hormonal treatment-related symptoms** | 2.6 (6.2) | 107 | 2.8 (4.1) | 56 | 2.8 (5.3) | 38 | 2.1 (3.4) | 48 | 4.4 (6.4) | 94 | 7.9 (7.9) | 47 | 5.2 (7.1) | 35 | 9.1 (7.9) | 42 |
| **Sexual activity** | 34.1 (19.5) | 105 | 32.1 (23.8) | 56 | 39.6 (18.6) | 37 | 29.2 (22.4) | 48 | 28.1 (19.6) | 92 | 18.8 (17.9) | 47 | 39.7 (22.1) | 34 | 18.7 (17.7) | 42 |
| **Sexual function (conditional)** | 78.3 (12.40 | 83 | 77.0 (13.7) | 42 | 81.9 (9.9) | 32 | 78.8 (12.0) | 33 | 73.5 (15.4) | 64 | 65.4 (15.9) | 27 | 75.3 (12.9) | 27 | 64.0 (17.4) | 20 |

|  | **After 6 months** |  |  |  |  |  |  |  |
| --- | --- | --- | --- | --- | --- | --- | --- | --- |
|  | **RP** |  | **EBRT** |  | **BT** |  | **HT** |  |
| **QLQ-C30 functioning scale** | **Mean (SD)** | **n** | **Mean (SD)** | **n** | **Mean (SD)** | **n** | **Mean (SD)** | **n** |
| **Global quality of life** | 78.5 (16.8) | 84 | 75.8 (13.9) | 44 | 75.0 (18.6) | 33 | 76.7 (16.5) | 39 |
| **Physical function** | 94.0 (10.4) | 85 | 91.4 (9.3) | 45 | 93.5 (10.6) | 33 | 91.2 (14.1) | 40 |
| **Role function** | 85.1 (21.5) | 83 | 83.0 (21.4) | 44 | 86.4 (21.8) | 33 | 83.8 (25.8) | 39 |
| **Emotional function** | 89.0 (17.3) | 84 | 88.4 (14.1) | 44 | 84.6 (21.1) | 33 | 88.2 (15.0) | 39 |
| **Cognitive function** | 90.9 (12.5) | 84 | 92.0 (14.6) | 44 | 88.4 (18.9) | 33 | 92.3 (14.7) | 39 |
| **Social function** | 89.1 (18.7) | 84 | 90.5 (17.8) | 44 | 86.9 (19.4) | 33 | 88.5 (23.3) | 39 |
| **QLQ-C30 symptom scales** |  |  |  |  |  |  |  |  |
| **Fatigue** | 17.4 (20.0) | 83 | 21.2 (19.4) | 44 | 18.5 (20..2) | 33 | 18.8 (19.3) | 39 |
| **Nausea and vomiting** | 0.6 (3.1) | 84 | 1.1 (4.2) | 44 | 1.5 (4.9) | 33 | 1.3 (4.5) | 39 |
| **Pain** | 5.2 (11.3) | 84 | 9.5 (15.4) | 44 | 11.6 (18.9) | 33 | 9.8 (18.6) | 39 |
| **Dyspnea** | 4.8 (12.9) | 83 | 8.3 (20.5) | 44 | 4.0 (11.0) | 33 | 6.0 (15.0) | 39 |
| **Insomnia** | 10.8 (18.1) | 83 | 23.5 (29.3) | 44 | 18.2 (27.8) | 33 | 23.9 (28.6) | 39 |
| **Appetite loss** | 4.0 (16.8) | 83 | 3.8 (12.9) | 44 | 2.0 (11.6) | 33 | 1.7 (7.4) | 39 |
| **Constipation** | 3.6 (12.7) | 84 | 6.1 (16.5) | 44 | 7.1 (16.2) | 33 | 7.7 (17.9) | 39 |
| **Diarrhea** | 4.4 (12.5) | 84 | 2.3 (8.5) | 44 | 9.1 (17.2) | 33 | 2.6 (11.8) | 39 |
| **Financial difficulties** | 4.4 (11.3) | 84 | 6.1 (18.0) | 44 | 7.1 (20.0) | 33 | 4.3 (11.3) | 39 |
| **QLQ-PR25 scales** |  |  |  |  |  |  |  |  |
| **Urinary symptoms** | 21.8 (15.8) | 84 | 22.0 (19.9) | 44 | 31.2 (23.8) | 33 | 20.9 (15.7) | 39 |
| **Incontinence aid (conditional)** | 19.0 (25.3) | 56 | 0.0 (0.0) | 4 | 20.0 (29.8) | 5 | 0.0 (0.0) | 6 |
| **Bowel symptoms** | 3.9 (10.1) | 84 | 4.4 (7.4) | 42 | 4.3 (10.9) | 31 | 4.2 (7.4) | 38 |
| **Hormonal treatment-related symptoms** | 7.7 (8.1) | 84 | 13.6 (11.8) | 42 | 7.8 (10.1) | 32 | 15.4 (11.4) | 39 |
| **Sexual activity** | 25.7 (18.6) | 83 | 16.3 (20.0) | 42 | 29.2 (18.0) | 32 | 9.4 (15.2) | 39 |
| **Sexual function (conditional)** | 58.2 (16.8) | 56 | 72.6 (16.6) | 19 | 69.5 (13.5) | 27 | 73.8 (18.8) | 14 |
